# Innate biosignature of treatment failure in human cutaneous leishmaniasis

**DOI:** 10.21203/rs.3.rs-4271873/v1

**Published:** 2024-05-02

**Authors:** Maria Adelaida Gómez, Ashton Trey Belew, Deninson Vargas, Lina Giraldo-Parra, David Rebellón-Sanchez, Theresa Alexander, Najib El Sayed

**Affiliations:** Centro Internacional de Entrenamiento e Investigaciones Médicas; University of Maryland; Centro Internacional de Entrenamiento e Investigaciones Médicas; Centro Internacional de Entrenamiento e Investigaciones Médicas - CIDEIM; Centro Internacional de Entrenamiento e Investigaciones Médicas; University of Maryland; University of Maryland

**Keywords:** Cutaneous leishmaniasis, treatment failure, antimony, RNA-Seq, innate immunity

## Abstract

The quality and magnitude of the immune and inflammatory responses determine the clinical outcome of *Leishmania* infection, and contribute to the efficacy of antileishmanial treatments. However, the precise immune mechanisms involved in healing or in chronic immunopathology of human cutaneous leishmaniasis (CL) are not completely understood. Through sequential transcriptomic profiling of blood monocytes (Mo), neutrophils (Nφ), and eosinophils (Eφ) over the course of systemic treatment with meglumine antimoniate, we discovered that a heightened and sustained Type I interferon (IFN) response signature is a hallmark of treatment failure (TF) in CL patients. The transcriptomes of pre-treatment, mid-treatment and end-of-treatment samples were interrogated to identify predictive and prognostic biomarkers of TF. A composite score derived from the expression of 9 differentially expressed genes (common between Mo, Nφ and Eφ) was predictive of TF in this patient cohort for biomarker discovery. Similarly, machine learning models constructed using data from pre-treatment as well as post-treatment samples, accurately classified treatment outcome between cure and TF. Results from this study instigate the evaluation of Type-I IFN responses as new immunological targets for host-directed therapies for treatment of CL, and highlight the feasibility of using transcriptional signatures as predictive biomarkers of outcome for therapeutic decision making.

## INTRODUCTION

Vector-borne infectious diseases cause more than 700,000 human deaths each year ^1^. Among the most impactful are those caused by viruses and intracellular protozoan parasites, including malaria, visceral and cutaneous leishmaniasis (CL), Chagas disease and dengue, which disproportionately affect the poor, and perpetuate the cycle of poverty and disease ^2^. CL annually affects about one million people in 92 countries ^3^. In the absence of vaccines and effective vector management, control of CL relies on treatment. However, the use and efficacy of most antileishmanials is limited by parenteral delivery, high levels of toxicity including hepato- and cariotoxicity ^4^, and increasing rates of treatment failure.

Despite WHO/PAHO guidelines favoring local treatments like thermotherapy or intralesional Sb^V^ therapy for uncomplicated CL, the vast majority of patients in endemic regions across the continent are still treated with systemic therapies, due to local protocol misalignment and limited access to technologies like thermotherapy. Furthermore, a high proportion of patients in the field (as much as 60% ^5^) are not eligible for local therapies, as they do not comply with the clinical criteria for use which are lesions in anatomical areas other than the face or near the joints, lesions < 3 cm of diameter, and patients presenting with a single lesion ^6^. Therefore, systemic antimonials remain the treatment of choice in clinical practice, which is reinforced by their lower cost and higher availability. This translates into a challenge for patients inhabiting rural and dispersed communities in most endemic areas, where access to medical attention is limited. Therefore, adherence to treatment and clinical follow-up are compromised, resulting in higher rates of treatment failure (TF). Consequently, there is an urgent need for predictive biomarkers of treatment outcome for these vulnerable patient populations, but most importantly, for better therapeutic interventions.

Clinical resolution of CL is accompanied by a reduction in parasite burden and yet, parasite elimination *per se* is not the sole determinant of healing. Parasite persistence has been documented following therapeutically achieved cure in more than 40% of patients infected with *Leishmania Viannia* species in Central and South America ^7–9^. Pathogenesis of CL is mediated by the immune and inflammatory responses; thus, resolution of disease and control of infection are intimately linked to the host response ^10^. Recent studies from our group and others are beginning to unravel the role of innate and adaptive immune components in TF of human CL upon treatment with antimonial drugs and miltefosine ^11–14^. We have shown that lesions of TF patients are characterized by sustained local inflammation mediated by heightened expression of pro-inflammatory chemokines and cytokines, predominantly associated with activation and migration of monocytes, polymorphonuclear cells, and T_H_1 CD4^+^ T cells ^13^. In addition, an environment of high cytolytic activity mediated by CD8^+^ T cells and subsequent induction of inflammation mediated by IL1β, has been shown in lesion biopsies of TF patients infected with *L.V*. *braziliensis* ^15^. Interestingly, the expression of inflammatory genes in peripheral blood mononuclear cells (PBMCs) of CL patients is modulated by *in vivo* exposure to antimonial drugs ^11^, as early as 30 minutes after intramuscular drug delivery. The dynamics of expression of some of those genes, including ones involved in monocyte and neutrophil chemotaxis and activation (*ccl2, cxcl2, cxcl3, cxcl8*), were related to the pharmacokinetics (PK) of plasma antimony concentrations. These observations suggest that drug-dependent modulation of inflammation, especially the immunomodulation of the innate responses, is a central component of healing in CL.

We hypothesized that a loop of sustained and deregulated inflammatory gene expression in innate cells leads to their continuous activation and recruitment to cutaneous lesions, promoting immunopathology and leading to TF in CL patients. Here, we present the first comprehensive transcriptomic profiling of peripheral blood monocytes (Mo), neutrophils (Nφ) and eosinophils (Eφ) of CL patients undergoing antileishmanial chemotherapy with meglumine antimoniate (Glucantime – GLUC). The profiles from each of the innate immune cell types interrogated here yielded clear signatures that distinguished TF patients from those who cured. Most prominent, and shared between the three cell types, was an enhanced Type I interferon (IFN) gene expression profile which comprised the hallmark signature of innate cells in patients with TF. A score derived from the expression levels of a subset of Type I IFN-inducible genes successfully classified cured and TF patients, even before initiation of treatment. Characterization of the innate immune response during the course of treatment allowed identification of host-specific prognostic signatures of the therapeutic response. This constitutes a solid first step toward the rational selection of host-targeted CL therapeutics that redirect inflammation and control pathology.

## RESULTS

### Study participants and samples

**Figure 1:**
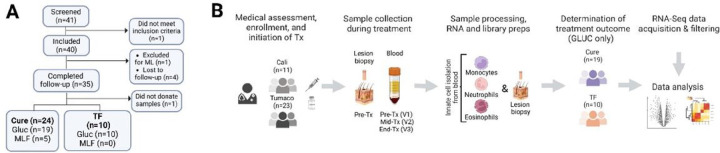
Participant recruitment and study design. Flow chart (**Panel A**) showing the recruitment of participants in the study ending with a total of 34 enrolled participants. Study design (**Panel B**) detailing participant enrollment, treatment, sample collection and analysis, and determination of treatment outcome.

Forty adult participants treated with antileishmanial therapy were included in this study, and recruited in our outpatient clinics, one in the South Pacific coast of Colombia in Tumaco, and another in the urban center of Cali. One patient was excluded due to progression to mucosal leishmaniasis, in accordance with the clinical protocol designed for this study (Fig. 1A). Of the remaining participants, 35 completed clinical follow-up. One participant did not donate blood or tissue samples and was therefore not included in the transcriptomic analyses. Most of the 34 enrolled participants were male of Afrocolombian descent, presented with ulcerated skin lesions, and most were infected with *L.V. panamensis*. Five patients were treated with miltefosine (MLF), all cured. Twenty-nine patients were treated with GLUC, 19 cured and 10 experienced TF. Due to the small sample size of MLF-treated patients and the absence of TF in this group, only samples from GLUC-treated patients (29 in total) were carried forward for transcriptomic analyses (Fig. 1B). Adherence to treatment for GLUC-treated patients was > 95% (measured as the proportion of ampoules received from those prescribed). Significant differences were noted in the age and ethnicity of patients who cured vs. patients with TF in the aggregated data of patients recruited in Cali and Tumaco (**Table S1a**).

Drug resistance and virulence of the infecting *Leishmania* strain may contribute to TF. In *L. V. braziliensis* and L. *V. guyanenesis* infections, virulence has been associated with the presence of the *Leishmania* RNA virus (LRV). To assess the impact of these variables, we evaluated the *Leishmania* isolates from study participants for susceptibility to pentavalent antimony and the presence of LRV. *Leishmania* strains were isolated from 24 patients (14 cures and 10 TF); isolates from the remaining 5 could not be propagated. As intracellular amastigotes, 10 strains were susceptible to GLUC, 12 were resistant (of which 5 were isolated from patients who were cured, and 7 from TF), and data was unavailable for 2 (**Fig. S1**). No statistically significant difference was found (Fisheŕs P = 0.23). All strains evaluated were negative for LRV. Together, these results rule out a significant contribution of virulence and drug resistance to the outcome of treatment in this cohort of patients.

### Global assessment of samples and transcriptomic profiles

**Figure 2:**
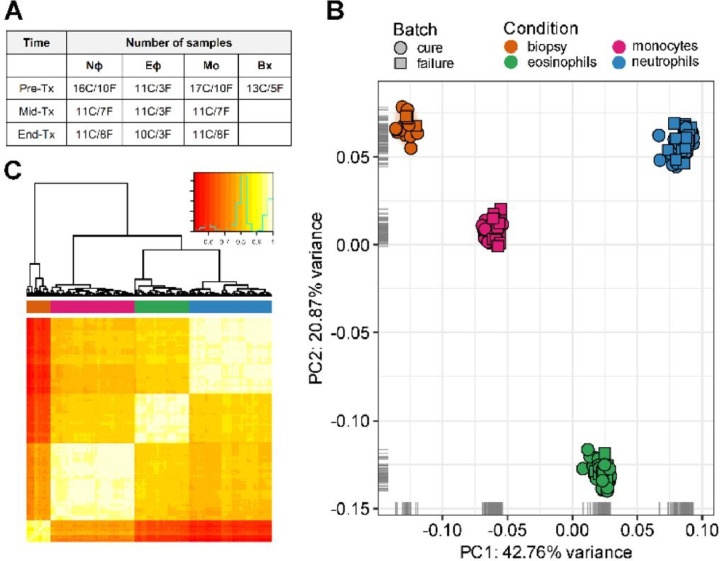
Global gene expression profiles of blood cells (Mo, Nφ, and Eφ) and lesion biopsies from study participants during the course of treatment. RNA-seq was carried out on biopsies collected pre-treatment and 3 types of leukocytes (Mo, Nφ, and Eφ) collected pre-treatment (Pre-Tx), mid-treatment (Mid-Tx), and at the end of treatment (End-Tx). C = cures, F= treatment failure (**Panel A**). A principal component analysis (PCA) plot (**Panel B**) and heatmap of hierarchical clustering analysis using pairwise correlations (**Panel C**) are shown for all samples. The analyses were performed using all annotated protein-coding genes following filtering for low counts, cpm, quantile normalization and log_2_ transformation. In the PCA plot, the first two principal components are shown on the X and Y axes, respectively, with the proportion of total variance attributable to that PC indicated. Each sample is represented as a single point with color indicating cell type and shape indicating treatment outcome. Colors along the top of the heatmap indicate the cell type. Colors within the inset of the heatmap represent correlation. The green line represents the frequency of correlation values.

Lesion biopsy samples were collected before initiation of treatment. Peripheral blood samples were obtained pre-treatment (Pre-Tx), mid-way through treatment (Mid-Tx, day 8), and at the end of treatment (End-Tx, day 20). Mo, Nφ, and Eφ were isolated from all blood samples and cDNA libraries were constructed and sequenced from a total of 186 samples (**Table S2_metadata**). Following low coverage filtering (**Fig. S2**), two samples were removed. For the remaining 184 samples (Fig. 2A), we used principal component analysis (PCA) and a correlation heatmap analysis to visualize the relationship between samples (Fig. 2). The PCA resulted in the expected grouping by cell type (Fig. 2B). A similar clustering was observed in hierarchical clustering analysis (Fig. 2C). These data support the quality, reproducibility and specificity of transcriptomes derived from the isolated blood cells from our patient cohort.

**Figure 3:**
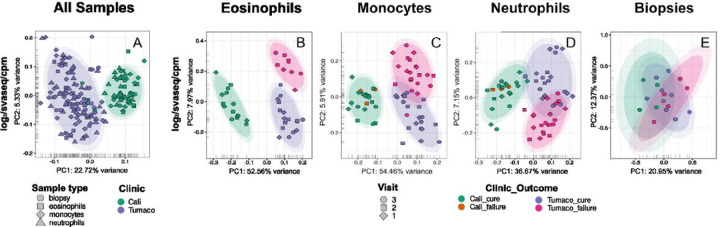
Assessment of outcome and clinic-associated batch effect. Principal Component Analysis (PCA) plots of the global gene expression profiles of all samples combined (**Panel A**) and individual blood cells (eosinophils, monocytes, and neutrophils) and lesion biopsies collected from study participants (**Panel B-E**) at two different clinics (one in Tumaco, the South Pacific coast of Colombia, and another in the urban center of Cali), and three visits (pre-treatment and twice during the course of treatment). Here, and based on the results shown in Fig. S3 we modeled the ‘clinic’ variable in SVA and used the surrogate variables-modified counts to generate the PCA plots. The analyses were performed using all annotated protein-coding genes following filtering for low counts, cpm normalization and log_2_ transformation. In the PCA plots, the first two principal components are shown on the X and Y axes respectively, with the proportion of total variance attributable to that PC indicated. Each sample is represented as a single point with color indicating either the clinic (Panel A), or clinic, treatment and outcome (Panels B-E). The inner and outer colored ellipses represent the 90% and 95% confidence intervals, respectively. These are only shown for classes containing more than 3 samples.

The use of two clinics for patient recruitment (one in Tumaco and another in Cali) necessitated the evaluation of the data to account for possible clinic-associated batch effects. When all samples were colored by clinic on the same PCA plot, no grouping by clinic was discernible within cell types (**Fig. S3, Panel A**); however, a grouping of samples by cell type revealed a significant amount of variance which separated Tumaco from Cali, confirming the presence of a batch effect attributable to the clinic (**Fig. S3, Panel B-D**). Similar analyses were performed evaluating the effect of ethnicity, without finding any substantial contribution of this variable to overall variability of the data (**Fig S3, Panels F-H**). To highlight this variability between clinics, we modeled the ‘clinic’ variable in SVA and used the surrogate variables-modified counts to generate the PCA plots, further supporting the hypothesis of a strong batch effect associated with the clinic (Fig. 3, **Panels A and B-D**).

To investigate the contribution of each potential variability-contributing metadata variable (such as clinic, donor, visit number, ethnicity or cell type), we performed a loadings analysis of the Surrogate Variables (SVs). First we performed a SV analysis (SVA) with cure/TF status as the variable of interest to calculate the SV loadings. Next, we calculated the F-statistic for each metadata variable (such as clinic location, donor, visit number and cell type) with respect to each SV (**Table S3**). This metric estimates if each factor is a good indicator of separation based on that SV loading, with higher F-statistic values indicating more between-class variation and less within-class variation. We found that the largest components of variance in the expression data were ‘cell type’ and ‘clinic’, followed by modest ‘donor/participant’ variability. After sub-setting to include only samples collected from Tumaco, we identified ‘cell type’ and ‘donor’ as variables contributing to expression variability (**Table S3**). Notably, we did not see a large contribution due to ‘visit number’ in any SV calculations, showing that the expression changes in the samples are modest between samples collected Pre-Tx, Mid-Tx or at End-Tx. SV1 and SV2 variability was dominated by the cell type, while SV3 through SV5 are representative of the donor and clinic (which are confounded). Visit number variability was not represented in any SV, indicating again that there is minimal variability in gene expression in samples collected at different time-points during treatment.

Based on the significant batch effect introduced by the patient recruitment clinic, and the skewed representation of cured patients recruited in Cali (9 of 10 CL patients recruited in this site cured), we excluded all samples obtained from patients in the Cali clinical site from the initial transcriptomic analyses and biomarker discovery. Therefore, transcriptomic variance associated with therapeutic outcome was analyzed on SVA-transformed data sets.

### Transcriptomes from lesion biopsies obtained pre-treatment, corroborate heightened tissue cytolytic activity in treatment failure patients

A recent comparative transcriptome analysis of Pre-Tx lesion biopsies from CL patients infected with *L.V. braziliensis* showed a gene signature of heightened cytolytic activity in lesions from TF patients compared to those who cured ^15^. To explore the congruity of those findings with infections with other *L. Viannia* species, we examined CL lesion transcriptomes in our study cohort. Overall, the transcriptomic profiles of lesion biopsies from patients who cured were indistinguishable from patients with TF (**Fig. S3E**), even following SVA (Fig. 3E). As expected, only few genes (n = 28) were differentially expressed (DE) (*P* < 0.05; |log_2_FC| ≥ 1) in lesion biopsies, 17 of which were up-regulated and 11 down-regulated in patients who went onto fail treatment, compared to cures (**Table S4a**). Notably, among up-regulated genes, a signature of increased cytolytic activity (*GZMB, NCR1, SH2D1B, PRF1, KLRC1, GNLY, FGFBP2, KIR2DL4, CCL3* and *CCL4*) was found in tissue samples from TF patients ((**Table S4b**), consistent with previous findings from TF patients infected with *L.V. braziliensis* ^15^. The minimal difference in the global transcriptomes of skin lesions from patients that cured and TF patients was expected, since bulk transcriptomes from complex multicellular tissues are often skewed to reflect the most abundant or transcriptionally active cells within the sample. Nevertheless, the fact that a clear transcriptomic signature of enhanced cytolytic activity was detected in TF suggests that systemic differences leading to the activation and/or recruitment of cytotoxic Natural Killer (NK) and CD8^+^ T cells could be contributing to this enhanced inflammatory state.

### Transcriptomic profiles of innate immune cells do not change over the course of treatment

Our understanding of the participation of innate immune responses in the outcome of antileishmanial therapy is almost exclusively limited to the role of macrophages as primary host cells for the parasite. However, mounting evidence shows that other innate cells including Nφ, and more recently Eφ and NK cells, participate in the inflammatory responses that contribute to CL immunopathology, and thus their functions are relevant to the outcome of treatment ^16^.

Among the hallmarks of innate immune cell functions are the velocity and robustness of their elicited responses, which in turn require tight mechanisms of control to avoid host injury. To explore the dynamics of these responses and their participation in therapeutic responsiveness, we analyzed samples collected from CL patients at three different visits: Pre-Tx, Mid-Tx and End-Tx. PCA plots of the transcriptomes of individual cell types did not reveal any significant clustering of samples based on visit (i.e. over the course of treatment) (**Fig. S4**). Thus, we opted to group transcriptome samples from all time points from each cell type, to provide increased robustness to DE analyses for identification of biomarkers and transcriptional signatures of cure and TF.

### Monocyte, neutrophil and eosinophil transcriptomes from CL patients who cure differ from those with TF

**Figure 4:**
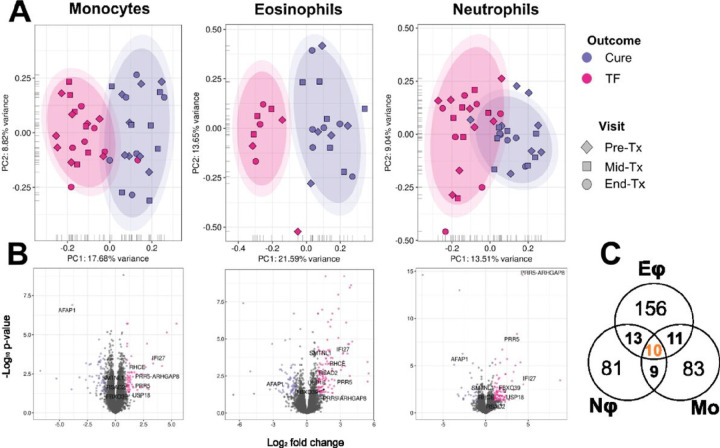
Global gene expression profiles of innate immune cells from cures and TF patients. PCA plots after batch correction estimated by SVA, of transcriptomes collected at all-time points from monocytes (Mo), eosinophils (Eφ) and neutrophils (Nφ) (**Panel A**). Volcano plots of differentially expressed (DE) genes for each cell type, with genes up-regulated in TF vs. cures colored pink, and those down-regulated in TF vs. cures labeled purple (**Panel B**). Venn diagram showing the intersection of common DE genes (P <0.05 and |log_2_FC| ≥ 1) and between cell types (either up- or downregulated) (**Panel C**). The inner and outer colored ellipses represent the 90% and 95% confidence intervals, respectively. These are only shown for classes containing more than 3 samples.

Examination of Nφ, Mo and Eφ transcriptomes showed a clear clustering of samples by treatment outcome, in each of the three cell types, revealing a distinct separation of samples along the first principal component (PC1) (Fig. 4A). On average, PC1 explained 18% of the variance across the three cell types. A DE analysis in each of three cell type transcriptomes revealed 3 to 4 times more significantly DE genes between cures and TF in Mo, when compared to Eφ and Nφ (**Table S5**), consistent with higher transcriptional activity of Mo. Those DE profiles are represented in the form of volcano plots (Fig. 4B). However, when a |log_2_FC| ≥ 1 threshold was established, a comparable number of DE genes was observed between cures and TF among the three cell types: 113 in Mo, 190 in Eφ, and 113 in Nφ (**Table S5**). Although more than 70% of significant DE genes with |log_2_FC| > 1 were unique to each cell type (Fig. 4C), gene enrichment analyses showed common significantly enriched features between the different innate cells (**Table S6**). Among up-regulated genes, enrichment of type I IFN responses was common to Mo, Eφ and Nφ from TF patients, and this was also supported by GSVA (**Table S7**). No similarities in gene enrichment analysis were found for down-regulated genes among cell types, with the exception of MHCII related genes (discussed below) (**Table S6**).

**Figure 5:**
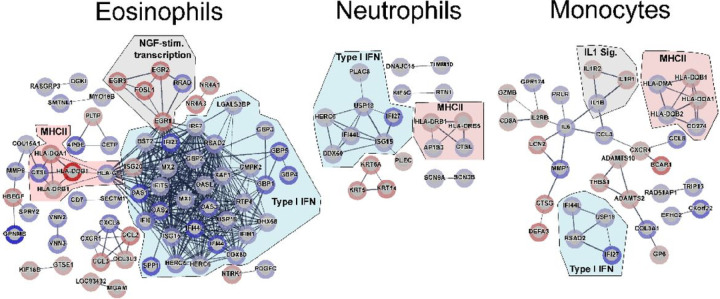
Network analysis of innate cell DE genes, contrasting cures vs. TF. Significantly DE genes (*P* < 0.05 and |log_2_FC| ≥ 1) were used as input for network analyses. STRING V11.5 was used to construct networks based on co-expression, databases and experiments terms, with line thickness representing confidence of the interaction. Enriched categories shown are those selected from KEGG, GO and Reactome terms with FDR<0.05 and strength >1. The blue border in nodes represents up-regulated genes in TF compared to cures; red borders depict downregulated genes. The intensity of the border color reflects the magnitude of the DE in our dataset. Genes belonging to the Type I IFN cluster are grouped under a blue shade, MHCII in red, and others in grey.

In Eφ, a robust induction of IFNα/β signaling was revealed by an up-regulated gene profile which included innate immune receptors, signaling molecules, transcription factors and regulators of signaling pathways (**Table S5**). The gene encoding IRF7 was up-regulated in TF Eφ, which together with IRF3, are the canonical transcriptional regulators of Type I IFNs ^17^. IFIH1 (gene encoding MDA5), a RIG-I-like receptor and cytoplasmic sensor of dsRNAs, was also up-regulated in TF. MDA5 has been demonstrated to be an amplifier of innate immune responses and associated with autoinflammation ^18^. Genes encoding downstream effectors (including *OAS1, OAS2, OAS3, OASL, BST2, MX1, MX2, IFI6, XAF1, GBP2*, and *IFI27*), and pathway regulators (*IFIT5, ISG15, RSAD2, USP18, HLA-G, DHX58,* and *DDX60*) were included in enriched gene categories in Eφ (Fig. 5), further substantiating a signature of enhanced Type I IFN responses in TF patients. Type I IFN response pathways were also enriched in Nφ transcriptomes from TF (**Table S6** and **Table S7**). Notably, genes encoding *HERC6, IFI44L, ISG15, USP18, IFI27* and *DDX60* (Fig. 5) were also expressed at higher levels in TF Nφ. Consistently, monocyte transcriptomes from TF patients were also enriched in mRNAs encoding Type I IFN-related genes, suggesting synergistic innate cell functions towards enhanced type I IFN inflammation in TF patients. Downregulation of IL1R1 and IL1R2 (decoy) receptors was observed in Mo from TF patients, and downregulation of molecules related to MHCII antigen presentation was found in all cell types from TF patients (**Table S5** and **Table S6**). Genes involved in wound healing and cell proliferation (*HBGEF, EGR1*, and *EGR3*) were found down-regulated in Eφ of TF patients. In Mo from TF patients, down-regulated antimicrobial peptide genes (including *CTSG, LTF, CAMP, DEFA3*, and *LCN2*) and immune receptor activity genes (including *IL2RB, IL1R2, HLA-DQA1, HLA-DQB1*, *IL1R1*, and *CXCR4*) were significantly enriched. Altogether, these results suggest that mechanisms underlying TF are associated with an enhancement of Type I IFN signaling, a dampening of antimicrobial effectors (antimicrobial peptides), and functions linking the innate and adaptive immune systems (antigen presentation).

In a complementary approach to DE analyses, we employed weighted gene co-expression network analysis (WGCNA) to identify co-expressed gene clusters associated with therapeutic response. The Mo data resulted in four gene modules with a significant association. The four modules comprised 478 genes altogether and were enriched in genes encoding ribosomal, mitochondrial, DNA nuclear activity and IFNα/β-inducible proteins (**Table S8, Figure S5**). In Nφ, three modules comprising a total of 581 genes were associated with therapeutic response and were enriched in cellular pathways similar to those found in our DE analyses: Type I IFN, MHC-II, DNA nuclear activity, glycolytic metabolism, cell migration, vesicular transport, cell death and the immunoproteasome. For Eφ, both associated modules comprised a total of 200 genes, with an enrichment of genes related to Type I IFN responses consistent with DE analysis, and Nφ and Mo WGCNA. Of the total genes contained within the significant modules from the three cell types, 36 were shared and all were up-regulated in TF. Among those, 24 were related to the type I IFN response (**Figure S5, Panel C**), and this was consistent with the DE analyses. Prominent in the WGCNA analyses were STAT1 and STAT2, two known transcription factors involved in Type I IFN signaling and expression of IFN-inducible genes. Both transcription factors were up-regulated in TF, supporting the coordinated and sustained Type I IFN effector functions elicited downstream of IFN receptor ligation. Together, WGCNA and DE analyses support the participation of a systemic pro-inflammatory environment sustained in TF, mediated by Type I IFNs.

### IFNα/β-inducible genes constitute a hallmark signature of TF in CL

**Figure 6:**
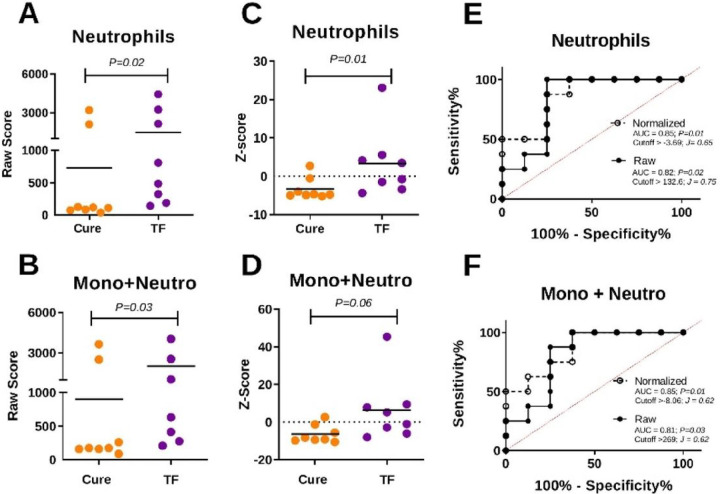
Performance of innate score for predicting TF. RPKM data of the 10 innate signature genes from Pre-Tx samples was used to construct neutrophil (panels A and C) and multi-cell (monocyte + neutrophil panels B and D) composite scores. Raw (A, B) and normalized scores (C, D) are shown. Statistical significance was evaluated by analysis of variance. P values from two-tailed analyses are shown. Representation of receiver operating characteristic (ROC) curves for raw (filled line and black circles) and normalized (dashed line and open circles) scores from neutrophils (E) and a combined score of monocytes plus neutrophils (F). ROC curves represent the area under the curve (AUC) of the false positive rate versus the true positive rate. P values, AUC, and cutoff values based on Youden’s J statistic are shown in the graph plots. Mono+Neutro: combined scores of monocytes and neutrophils.

Based on the functional commonalities between the cell-specific transcriptional profiles, we recognized a common innate gene signature, from which we were able to identify biomarkers that predict TF. From the gene lists derived from our WGCNA and DE analyses, we selected the 10 common DE genes in Mo, Nφ and Eφ: *IFI44L, IFI27, PRR5, PRR5-ARHGAP8, RHCE, FBXO39, RSAD2, SMTNL1, USP18*, and *AFAP1*. Each of those common genes were up-regulated in TF, with the exception of *AFAP1*, which was down-regulated. We therefore constructed a predictive composite score for each patient based on this innate gene signature (excluding *AFAP1* because it was downregulated in TF). RPKM values were extracted from all Pre-Tx samples for each cell type. Those were selected for their predictive value in guiding early therapeutic interventions. RPKM values were used to construct raw and normalized scores (Z-score), the latter to account for possible outliers within groups. Both raw and Z-scores were higher in TF patients, and this difference was statistically significant for the scores derived from Nφ, and for the Mo + Nφ composite score (Fig. 6A–C and **Fig. S6**). Consistently, raw and Z-scores from Pre-Tx Nφ samples and the Mo + Nφ scores, were significantly predictive of the therapeutic response (Fig. 6E, F).

We next applied a machine learning approach to carry out a comprehensive analysis of all data on hand and complement our focused DE analyses. The expression matrices of all samples obtained from both clinics (Tumaco and Cali) were split into 10 rounds of training (40%) and testing sets (60%), through random partitioning and cross-validation. The training sets were used to generate K nearest neighbors (KNN), logistic regression (GLM), gradient boost (XGBoosted GLM), and random forest models ^19^. Following an evaluation of the resulting models by comparing the predictions of the training data to the known clinical outcomes (**Table S9**), we used those models to predict clinical outcomes in the test partition and evaluated their performance with an emphasis on a dataset that only included Pre-Tx samples as these would be the most translatable for clinical and therapeutic decision making. The GLM model appeared to best predict the clinical outcome with good overall performance (accuracy 0.73/0.89, sensitivity 0.74/0.95, specificity 0.95/0.80, and AUC 0.6/0.85; each pair of values is the mean of Pre-Tx / mean of all visits) (**Table S9**). As expected, addition of samples from subsequent visits enhanced the performance of all models (**Table S9**).

## DISCUSSION

The response to antimicrobial chemotherapy has been primarily believed to depend on the drug susceptibility of the etiological agent, patient adherence to the therapeutic scheme, and intrinsic differences in drug exposure (PK). It is known that immunosuppression negatively impacts the efficacy of antileishmanials, both in murine models of infection ^20–24^ and in humans ^25,26^. However, approximately 25% of immunocompetent individuals present with TF in controlled clinical trials ^27–29^. In this study we elucidated a central role for Type I IFN innate inflammatory responses in TF during systemic treatment with meglumine antimoniate (Glucantime^®^).

Type I IFNs (IFNα and IFNβ) are rapidly induced during viral infections, and are central to the antiviral response ^17^. However, their role in infections with intracellular bacteria or protozoan parasites remains elusive. A low dose of IFNβ protected mice from progressive CL caused by *L. major* ^30^, and this was related to induction of iNOS, NK cytotoxicity, and early production of IFNγ ^31^. In visceral leishmaniasis (VL) caused by *L. donovani*, IFNα/β acts as an upstream suppressor of anti-parasitic T_H_1 cells, and IFNAR1 −/− mice better control infection compared to wild type ^32^. These results provide evidence of both protective and pathogenic roles of Type I IFNs in leishmaniasis, which are likely dependent on the IFNα/β concentration and downstream regulation of the response. In human PBMCs, pharmacological blocking of IFNα/β resulted in antigen-specific increase of IFNγ production, and this was reverted by inhibition of MHCII (HLA-DR) antigen presentation ^32^. Interestingly, heightened expression of IFNα/β-stimulated genes (ISGs) in Mo, Nφ and Eφ of TF patients was consistently accompanied by significant dampening of MHCII gene expression. This suggests that, similar to what occurs in VL, an impaired protective immunity mediated by type I IFNs via antigen presenting cells, could be occurring in CL.

Recent evidence shows that Type I IFNs promote pathogenesis and severity of *M. tuberculosis* (MTB) infection in both mice and humans ^33,34^. This was associated with dampening of protective IL-1 signaling via eicosanoid imbalance. In monocytes from TF, high type I IFN-inducible gene expression was accompanied by repression of IL1R1 and IL1R2 (decoy receptor), and increased IL1β. Downregulation of IL1R1, even in the context of up-regulated IL1β, suggests that the cross-balance of Type I IFNs and IL1 is not only relevant to the pathogenesis and severity of TB, but also to the outcome of CL treatment.

Severity and tissue damage during infectious diseases is often mediated by immunopathology caused by exacerbated and uncontrolled inflammatory responses. During viral infections, IFNα/β promote CD8^+^ T cell longevity and clonal expansion ^35^, as well as NK cell functions ^17^. Notably, Pre-Tx lesion biopsies from TF patients exhibited a transcriptional profile compatible with enhanced cytolytic activity mediated by CD8^+^T and NK cells, similar to what observed in TF patients infected with *L.V. braziliensis* ^15^. It is plausible that the heightened Type-I IFN responses, mediated by systemic innate inflammatory cells, could contribute to skin immunopathology driven by CD8^+^T and NK cells in lesions of CL patients who do not respond to treatment.

The mechanisms by which IFNα/β-inducible genes are modulated during *Leishmania* infection remain unknown, and constitute part of our ongoing investigations. In *L. Viannia* infections, expression of IFNα/β and its contribution to disease severity has been proposed to be mediated by the *Leishmania* RNA virus (LRV). A systematic evaluation of the presence of LRV in more than 100 *L.V. panamensis* clinical strains did not show evidence of the presence of LRV in this species ^36^. This was consistent with the absence of LRV in all *L.V. panamensis*, as well as in *L.V. braziliensis* clinical isolates from our study participants, ruling out any contribution of LRV to the Type I IFN signature of innate cells observed in TF patients. We have previously shown that *L.V. panamensis* induces TLR4 gene expression as early as 8h after interaction with human monocyte-derived macrophages ^37^. Intracellular parasite survival and TNFα production were found to be dependent on TLR4 ^37^. Furthermore, using RNA-seq, we have shown induction of a Type-I IFN signature in human PBMCs, occurring as early as 24h after *L.V. panamensis* infection with strains associated with chronic CL, and not with those causing self-healing disease ^38^. These previous findings lead us to hypothesize that rapid and, likely, strain-specific TLR engagement by Leishmania could induce a rapid (8h) IFNα/β gene expression, leading to a second wave of IFNα/β-inducible genes as early as 24h post infection.

The slow discovery pipeline for novel antimicrobials, and especially for those causing neglected tropical diseases, has driven the development of innovative approaches such as host-directed therapies (HDTs). The basis of HDTs relies on detailed understanding of the role of host responses in pathogenesis and clinical outcome of infections. However, HTDs for leishmaniasis, including immunomodulation, have often been based on knowledge of the contribution of immune responses to disease in animal models, resulting in failed clinical trials ^39–42^. Understanding the innate factors driving therapeutic healing of CL offers a unique opportunity for rational identification of HTDs that optimize available therapeutic regimens, and can capitalize on past and current pharmaceutical developments in modifiers of innate immune functions. Interestingly, antibody-mediated blocking of IFNαR in mice, or the use of FDA-approved ruxolitinib (a small molecule inhibitor of JAK1 and JAK2), synergized with amphotericin B to control *L*. *donovani* infection ^32^. Our data suggest that modulation of Type I-IFN responses is a likely target for host-directed therapy in CL caused by *L. Viannia*.

The high frequency and severity of adverse events and of TF during antileishmanial treatment demands stratification of therapeutic interventions to populations where they will be most effective. Results from this study revealed a transcriptional Type-I IFN innate signature of TF. This signature allowed construction of a composite score, with significant specificity and sensitivity to predict TF before initiation of treatment. Although our sample size presents an evident limitation for successful implementation of machine learning techniques, GLM models were good predictors of outcome, albeit using the top 3000 most variable genes. The area under the curve (AUC) achieved using our composite scores, which incorporate 9 genes, closely mirrored the AUCs obtained with machine learning models that included all 3000 genes. This similarity not only underscores the robustness of our selected genes but also validates the effectiveness of our hallmark in capturing the essential genomic signatures. Notably, the score derived from Nφ, and the composite score from Mo + Nφ were both predictive of TF. That Nφ are the most abundant white blood cells in blood, and monocytes one of the most transcriptionally active, supports the likelihood of developing whole blood tests for future validation, which also facilitate access in remote rural populations. Similar scoring systems have assisted screening in other immunological systems. IFN-scores based on gene expression profiles of Type-I IFN stimulated genes have been used as screening tools for monogenic interpheronopathies, and to stratify patients with systemic lupus erythematosus ^43,44^. Genes reported in these scoring systems often differ between diseases. However, four of the ten genes that composed our IFN-signature (*IFI27, IFI44L, USP18* and *RSAD2*), have been consistently reported as signature members in other autoinflammatory diseases, and used for clinical assessments ^43,45^.

Personalized medicine is anticipated to be restricted to developed countries widening the disparity between “the rich and the poor” ^46^. Technology-driven research, systems medicine and genetic knowledge should reduce health care disparities rather than exacerbating them. Our study provides a solid first step towards validation and implementation of personalized medicine for CL, one of the most neglected tropical infectious diseases of global importance.

## MATERIALS AND METHODS

### Ethics statement.

This study (IRB code #1273) was approved and monitored by the Institutional Review Board for ethical conduct of research involving human subjects of the Centro Internacional de Entrenamiento e Investigaciones Médicas (CIDEIM) in accordance with national (Resolution 008430, República de Colombia, Ministry of Health, 1993) and international (Declaration of Helsinki and amendments, World Medical Association, Fortaleza, Brazil, October 2013) guidelines. All individuals voluntarily participated in the study and written informed consent was obtained for each participant.

### Study design and subjects.

This study was designed to identify host biomarkers and innate immune functions that participate in the response to antileishmanial treatment in CL patients. Transcriptional profiling of innate immune cells and lesion biopsies was conducted. Adult patients (18 to 60 years of age) with parasitological diagnosis of active CL with a time of evolution < 6 months, and without apparent immune deficiencies (negative HIV test, no evidence of immunological disorder or treatment with medication having immunomodulating effects), who received standard-of-care treatment with Glucantime (GLUC, 20 mg/kg/day for 20 days) or miltefosine (MLF, 1.8–2.5 mg/kg daily dose for 28 days) were included in this study. Treatment outcome was evaluated at week 13 following initiation of treatment for GLUC and at week 26 for MLF. Cure was defined as complete re-epithelization and absence of inflammatory signs for all lesions. TF was defined as incomplete re-epithelization and/or the presence of induration, raised borders, or redness in any lesion; reactivation of the original lesion(s); or the appearance of new lesions.

### Skin lesions biopsies samples.

Skin lesion punch biopsies were obtained before initiation of treatment. Biopsy punches of 3 mm were obtained under local anesthetic, taking into account the following ratio: 1/3 of healthy skin and 2/3 of the edge of the lesion (the indurated edge, which does not include necrotic tissue). Skin biopsies were immediately stored in 1 mL Allprotect^®^ (Qiagen). Samples were equilibrated overnight at 4°C and then stored at −20°C until processing ^47^.

### Isolation of monocytes, neutrophils and eosinophils from peripheral blood samples.

Ninety mL of whole blood anticoagulated with EDTA was obtained from each patient. PBMCs and polymorphonuclear leukocytes (PMNs) were isolated by centrifugation over a Polymorphprep^™^ (Axis-Shield) gradient according to the manufacturer’s instructions. CD14 + Mo were purified from PBMCs using the CD14 microbeads ultrapure kit (Milteny Biotec) coupled to magnetic cell sorting (MACS). CD16 + Nφ were purified from PMNs using CD16 microbeads human kit (Milteny Biotec) and Eφ were obtained by negative selection using the eosinophil isolation kit human (Milteny Biotec). Cells were washed with cold PBS, precipitated by centrifugation, and the cell pellet was resuspended in 100–200 μL of RNAlater (Ambion) and stored at −80°C for later use. Purity of isolated cell populations was evaluated by flow cytometry and light microscopy. Samples were used only when purity was > 98%.

### Leishmania strains, typing and drug susceptibility testing.

*Leishmania* isolates were obtained from all patients and propagated in Senekjiés biphasic blood agar and immediately stored in liquid nitrogen until use. Strains were typed by immunoreactivity to monoclonal antibodies as previously described ^48^. Drug susceptibility of intracellular amastigotes was estimated by evaluation of % parasite survival in PMA-differentiated U-937 cells after exposure to pentavalent antimony (SbV) at a final concentration of 32 μg/mL, compared to control without drug exposure. *Leishmania* strains were defined as Sb-resistant when percent reduction of the parasite burden after drug exposure was < 78%. Susceptibility cutoff was defined based on a panel of well-characterized clinical isolates presenting with intrinsic resistance or susceptibility to SbV ^49,50^.

### RNA isolation and cDNA synthesis.

RNA isolation from purified cell populations (Mo, Nφ and Eφ) stored in RNAlater was performed using TRIzol^™^ (Invitrogen, USA), followed by RNA cleanup with RNeasy Mini Kit columns (Qiagen, USA). Isopropanol/water (1:1) was used for RNA precipitation. RNA isolation from lesion biopsies was performed by tissue disruption, homogenization and extraction using TRIzol^™^ reagent as previously described ^47^. RNA integrity was assessed using an Agilent 2100 bioanalyzer. For RNA-seq, poly(A)-enriched cDNA libraries were generated using the Illumina TruSeq v2 sample preparation kit (San Diego, CA) and checked for quality and quantity using bioanalyzer and quantitative PCR.

### RNA-seq data generation, preprocessing, and quality trimming.

Single and paired-end reads were obtained on an Illumina NovaSeq 6000 at the Genetic Resources Core Facility, Johns Hopkins Department of Genetic Medicine, Baltimore, MD; or on an Illumina HiSeq1000 at the Brain & Behavior Institute - Advanced Genomic Technologies Core (BBI-AGTC) at the University of Maryland, College Park, MD. Trimmomatic ^51^ was used to remove Illumina adapter sequences, discard reads shorter than 40 nucleotides, and trim any 4 nucleotide rolling window with a mean Phred quality score less than or equal to 20. Sequence quality metrics were assessed using FastQC (http://www.bioinformatics.babraham.ac.uk/projects/fastqc/). Raw data and project information available via NCBI-dbGaP study ID # 38338; phs003545.v1

### Mapping cDNA fragments and abundance estimation.

Reads were aligned against the human (hg38 revision 100), *L.V. panamensis* (TriTrypDb release 36), and *L.V. braziliensis* (release 26) genomes with HISAT2 (2.1.0) ^52^ using the default parameters. The resulting accepted hits and mapped reads were sorted and indexed via SAMtools ^53^ and passed to HTSeq ^54^ for generating count tables.

### Global data assessment, visualization and differential expression analysis.

Biological replicates and batch effects were assessed and visualized using an R package, hpgltools (https://github.com/elsayed-lab/hpgltools), developed and maintained in the El-Sayed lab. Normalized data were visualized using log_2_ transformed counts per million (CPM) reads following filtering to remove low counts (defined as any gene with a sum less than twice the number of samples or when all samples had fewer than 2 counts). Samples in which fewer than 11,000 genes were observed in a non-zero genes plot, were removed. Following data filtration, visualizations were performed to observe the sample relationships; these included density plots, boxplots of depth, coefficient of variance, hierarchical clustering analyses based on Pearson’s correlation coefficient and Euclidean distance, variance partition analyses, and principal component analyses (PCA) before and after normalization. Several combinations of normalization and batch adjustment strategies were evaluated along with surrogate variable estimation via SVA ^55^. Samples were queried via cure/TF status, visit number, clinic, and cell type in order to calculate the surrogate variable (SV) loadings, and the F-statistic was calculated for each variable with respect to each SV.

Differential expression analyses were performed using a single pipeline which performed all pairwise comparisons using the Bioconductor packages: limma ^56^, edgeR ^57^, DESeq2 ^58^, EBSeq ^59^, and an explicitly basic analysis (using only Log_2_CPM values). In each case (except EBSeq and the basic analysis), the surrogate variable estimates provided by SVA were used to adjust the statistical model in an attempt to address the batch/surrogate effects. Each contrast was evaluated in context of its agreement with other methods, but the interpretations were primarily informed by the DESeq2 results. Detailed information on the analytical pipeline and scripts is available in Supplementary Material. Genes with significant changes in abundance (|Log_2_ fold change| ≥ 1 and false discovery rate adjusted *P* values ≤ 0.05) were passed to gProfileR ^60^ and GSVA ^61^. Gene ontology analyses were supplemented with manual data curation. Network analyses were performed with STRING 11.0 ^62^. Simultaneously, gene set variation analysis (GSVA) was performed to produce an enrichment score against the mSigDB ^63^ datasets (C2, C7, and H) on a per-sample basis. These scores were passed to limma to evaluate the difference in GSVA score distributions for each gene set in the samples. Results from limma were then filtered according to log_2_ fold change, adjusted *P* value, and maximum GSVA score mean.

### Detection of virus sequences.

Kraken 2, with a supplemented version of its viral database ^64^, was used to check each sample specifically for the *Leishmania* RNA virus (LRV), as well as any putative viral reads. Confirmation of LRV absence was performed by qRT-PCR as described ^36^.

### Weighted gene co-expression network analysis (WGCNA).

WGCNA co-expression networks were generated and examined using low-count filtered, SVA-adjusted, RPKM (Reads per kilobase per million) by average CDS length, log_2_-transformed counts as input. Pairwise Pearson correlations between each gene pair were calculated and transformed into a signed adjacency matrix using the minimum power that resulted in a scale-free R^2^ fit of 0.8. The resulting modules and associated eigengenes were produced via the default correlation matrix blockwise module detection methods from WGCNA. Module scores by sample were queried against the metadata factors (cure and TF) via Pearson’s correlations and scored using the *P* value metrics provided by WGCNA. The eigengenes were extracted from modules with scores deemed significant, manually examined, and passed to gene set enrichment methods. Modules significantly associated with the outcome and containing more than 1,000 genes were excluded from the analyses.

### Composite scores.

Pre-Tx RPKM values were used to construct raw and normalized (Z-score) composite scores. Selection of genes that constitute each individual composite was based on the DE genes between cures and TF that were common among Nφ, Eφ, and Mo (signature genes). Raw scores were calculated per patient, as the sum of RPKM values of the signature genes in each cell type (Eq. 1). A normalized score was also computed based on the sum of normalized RPKM values (Eq. 2). For raw and normalized scores, “I” indexes genes and “k” patients.

For each cell type:

equation 1) $$(\mathrm{rawscore}{)}_{k}=\sum_{i=1}^{n}\;RPKM_{ik}$$

equation 2) $$(\mathrm{normalizedscore}{)}_{k}=\sum_{i=1}^{n}\frac{RPKM_{ik}-{\overset{‾}{x}}_{i}}{\sigma_{i}}$$

Where

$${\overset{‾}{x}}_{i}=\frac{1}{m}\sum_{k=1}^{m}RPKM_{ik}\;\text{and}$$

$$\sigma_{i}=\sqrt{\frac{1}{m}\sum_{k=1}^{m}{\left(RPKM_{ik}-{\overset{‾}{x}}_{i}\right)}^{2}}$$

Receiver operating characteristic (ROC) curves were used to explore the predictive potential of raw scores and Z-scores to discriminate therapeutic cure and failure. Sensitivity and specificity parameters were calculated for scores. Youden’s J statistic was used to define cutoff values.

### Machine learning models.

A series of machine learning models were generated and examined via the caret R package ^19^ to create transcriptome-informed classifiers of patients likely to cure or fail treatment. Initial expression sets were selected to include all data from innate cells from patients recruited in Cali and Tumaco, or data only from Tumaco patients. The starting data were log_2_-transformed CPM values, normalized, filtered to exclude genes with CV < 0.1, centered, filtered to exclude genes with correlations ≥ 0.95. The most variable 3,000 genes were then selected. The remaining data was split into training (0.4) and testing (0.6) sets 10 times. The training datasets were used to create k-nearest neighbor, random forest, GLM, and gradient boost models with an arbitrarily chosen mix of bootstrap and CV sampling. The test partitions were evaluated for accuracy and sensitivity/specificity with respect to the known outcome of each patient.

### Statistical analyses.

For the exploration and description of the sociodemographic and clinical variables, univariate analyses were performed. Categorical variables were described with frequencies and percentages. Quantitative variables were described as means (± SD) or medians (IQR) according to the distribution of the data. For the comparison of qualitative variables, Fisher’s exact test or the chi2 test was used according to the distribution of the data. Fisheŕs test was also used for comparison of tables larger than 2×2 ^65^. Quantitative variables were compared using t-test or U-Mann-Whitney tests. Normality was determined with qq plots and the Shapiro wilk test. In all analyses *P*-values < 0.05 were considered significant. Statistical analysis was performed using GraphPad Prism version 9 and R version 4.1.3.

## Data Availability

All sequence data is publicly available at NCBI-dbGaP study ID # 38338; phs003545.v1

## References

[1] WHO (2020) Vector-borne diseases. https://www.who.int/news-room/fact-sheets/detail/vector-borne-diseases

[2] WHO/TDR. Global Report for Research on Infectious Diseases of Poverty (2012)

[3] WHO, Leishmaniasis (2020) Status of endemicity of cutaneous leishmaniasis 2020. https://apps.who.int/neglected_diseases/ntddata/leishmaniasis/leishmaniasis.html

[4] Oliveira LF (2011) Systematic review of the adverse effects of cutaneous leishmaniasis treatment in the New World. Acta Trop 118:87–96 21420925 10.1016/j.actatropica.2011.02.007

[5] Uribe-Restrepo AF, Prieto MD, Cossio A, Desai MM (2019) Del Mar Castro, M. Eligibility for local therapies in adolescents and adults with cutaneous leishmaniasis from southwestern Colombia: A cross-sectional study. Am J Trop Med Hyg 100:306–310 30628567 10.4269/ajtmh.18-0643 PMC6367628

[6] PAHO. Guideline for the Treatment of Leishmaniasis in the Americas (2022) 10.37774/9789275125038

[7] Rosales-Chilama M (2015) Parasitological Confirmation and Analysis of Leishmania Diversity in Asymptomatic and Subclinical Infection following Resolution of Cutaneous Leishmaniasis. PLoS Negl Trop Dis 9:1–20 10.1371/journal.pntd.0004273 PMC4684356 26659114

[8] Vergel C (2006) Evidence for Leishmania (Viannia) parasites in the skin and blood of patients before and after treatment. J Infect Dis 194:503–511 16845635 10.1086/505583

[9] de Camera P (2006) Haematogenous dissemination of Leishmania (Viannia) braziliensis in human American tegumentary leishmaniasis. Trans R Soc Trop Med Hyg 100:1112–1117 16765391 10.1016/j.trstmh.2006.02.014

[10] Scott P, Novais FO (2016) Cutaneous leishmaniasis: Immune responses in protection and pathogenesis. Nat Rev Immunol 16:581–592 27424773 10.1038/nri.2016.72

[11] Gómez MA (2021) Immuno-pharmacokinetics of Meglumine Antimoniate in Patients with Cutaneous Leishmaniasis Caused by Leishmania (Viannia). Clin Infect Dis 72:E484–E492 32818964 10.1093/cid/ciaa1206 PMC8130027

[12] Del Castro M (2017) Pharmacokinetics of miltefosine in children and adults with Cutaneous leishmaniasis. Antimicrob Agents Chemother 61:1–11 10.1128/AAC.02198-16 PMC5328512 27956421

[13] Navas A (2020) Profiles of local and systemic inflammation in the outcome of treatment of human cutaneous leishmaniasis caused by L. Viannia. Infect Immun 88:1–12 10.1128/IAI.00764-19 PMC7035935 31818959

[14] Kip AE (2015) Systematic review of biomarkers to monitor therapeutic response in leishmaniasis. Antimicrob Agents Chemother 59:1–14 25367913 10.1128/AAC.04298-14 PMC4291336

[15] Amorim CF (2019) Variable gene expression and parasite load predict treatment outcome in cutaneous leishmaniasis. Sci Transl Med 11:1–9 10.1126/scitranslmed.aax4204 PMC7068779 31748229

[16] Campos TM (2020) Granzyme B Produced by Natural Killer Cells Enhances Inflammatory Response and Contributes to the Immunopathology of Cutaneous Leishmaniasis. J Infect Dis 221:973–982 31748808 10.1093/infdis/jiz538 PMC7050991

[17] Mcnab F, Mayer-barber K, Sher A, Wack A, Garra AO (2020) Type I interferons in infectious disease. Nat Rev Immunol 15:87–103 10.1038/nri3787 PMC7162685 25614319

[18] Gregorio A, Junior D, Sampaio NG, Rehwinkel J (2019) A Balancing Act: MDA5 in Antiviral Immunity and Autoinflammation. Trends Microbiol 27:75–85 30201512 10.1016/j.tim.2018.08.007 PMC6319154

[19] Kuhn M (2008) Building Predictive Models in R Using the caret Package. J Stat Softw 28:1–2627774042

[20] Murray HW, Delph-Etienne S (2000) Roles of endogenous gamma interferon and macrophage microbicidal mechanisms in host response to chemotherapy in experimental visceral leishmaniasis. Infect Immun 68:288–293 10603400 10.1128/iai.68.1.288-293.2000 PMC97133

[21] Murray HW, Jungbluth A, Ritter E, Montelibano C, Marino MW (2000) Visceral leishmaniasis in mice devoid of tumor necrosis factor and response to treatment. Infect Immun 68:6289–6293 11035737 10.1128/iai.68.11.6289-6293.2000 PMC97711

[22] Murray HW, Montelibano C, Peterson R, Sypek JP (2000) Interleukin-12 regulates the response to chemotherapy in experimental visceral Leishmaniasis. J Infect Dis 182:1497–1502 11023473 10.1086/315890

[23] Murray HW, Nathan CF (1999) Macrophage microbicidal mechanisms in vivo: reactive nitrogen versus oxygen intermediates in the killing of intracellular visceral Leishmania donovani. J Exp Med 189:741–746 9989990 10.1084/jem.189.4.741 PMC2192937

[24] Murray HW, Oca MJ, Granger AM, Schreiber R (1989) D. Requirement for T cells and effect of lymphokines in successful chemotherapy for an intracellular infection. Experimental visceral leishmaniasis. J Clin Invest 83:1253–1257 2539396 10.1172/JCI114009 PMC303815

[25] Laguna F (2003) Treatment of leishmaniasis in HIV-positive patients. Ann Trop Med Parasitol 97(Suppl 1):135–142 14678640 10.1179/000349803225002606

[26] Laguna F (2003) Amphotericin B lipid complex versus meglumine antimoniate in the treatment of visceral leishmaniasis in patients infected with HIV: a randomized pilot study. J Antimicrob Chemother 52:464–468 12888588 10.1093/jac/dkg356

[27] Palacios R, Osorio LE, Grajales LF, Ochoa MT (2001) Treatment failure in children in a randomized clinical trial with 10 and 20 days of meglumine antimonate for cutaneous leishmaniasis due to Leishmania viannia species. Am J Trop Med Hyg 64:187–193 11442216 10.4269/ajtmh.2001.64.187

[28] Soto J, Toledo J, Vega J, Berman J (2005) Short report: efficacy of pentavalent antimony for treatment of colombian cutaneous leishmaniasis. Am J Trop Med Hyg 72:421–422 15827279

[29] Velez I (2010) Efficacy of miltefosine for the treatment of American cutaneous leishmaniasis. Am J Trop Med Hyg 83:351–356 20682881 10.4269/ajtmh.2010.10-0060 PMC2911184

[30] Mattner J (2004) Protection against progressive leishmaniasis by IFN-beta. J Immunol 172:7574–7582 15187137 10.4049/jimmunol.172.12.7574

[31] Diefenbach A (1998) Type 1 interferon (IFNalpha/beta) and type 2 nitric oxide synthase regulate the innate immune response to a protozoan parasite. Immunity 8:77–87 9462513 10.1016/s1074-7613(00)80460-4

[32] Kumar R (2020) Type I Interferons Suppress Anti-parasitic Immunity and Can Be Targeted to Improve Treatment of Visceral Leishmaniasis. Cell Rep 30, 2512–2525 e9 32101732 10.1016/j.celrep.2020.01.099 PMC7981274

[33] Mayer-Barber KD (2014) Host-directed therapy of tuberculosis based on interleukin-1 and type I interferon crosstalk. Nature 511:99–103 24990750 10.1038/nature13489 PMC4809146

[34] Berry MPR (2010) An interferon-inducible neutrophil-driven blood transcriptional signature in human tuberculosis. Nature 466:973–977 20725040 10.1038/nature09247 PMC3492754

[35] Kolumam GA, Thomas S, Thompson LJ, Sprent J, Murali-Krishna K (2005) Type I interferons act directly on CD8 T cells to allow clonal expansion and memory formation in response to viral infection. J Exp Med 202:637–650 16129706 10.1084/jem.20050821 PMC2212878

[36] Rosales-Chilama M, Oviedo MY, Quintero YK, Fernandez OL, Gomez MA (2023) Leishmania RNA Virus Is Not Detected in All Species of the Leishmania Viannia Subgenus: The Case of L. (V.) panamensis in Colombia. Am J Trop Med Hyg 108:555–560 36716739 10.4269/ajtmh.22-0551 PMC9978567

[37] Gallego C, Golenbock D, Gomez MA, Saravia NG (2011) Toll-like receptors participate in macrophage activation and intracellular control of Leishmania (Viannia) panamensis. Infect Immun 79:2871–2879 21518783 10.1128/IAI.01388-10 PMC3191987

[38] Gomez MA (2021) Early Leukocyte Responses in Ex-Vivo Models of Healing and Non-Healing Human Leishmania (Viannia) panamensis Infections. Front Cell Infect Microbiol 11:687607 34557423 10.3389/fcimb.2021.687607 PMC8453012

[39] Castro MDM (2022) Pentoxifylline in the Treatment of Cutaneous Leishmaniasis: A Randomized Clinical Trial in Colombia. Pathogens 11:378 35335703 10.3390/pathogens11030378 PMC8949591

[40] Pinart M (2020) Interventions for American cutaneous and mucocutaneous leishmaniasis. Cochrane Database Syst Rev 8:CD004834 10.1002/14651858.CD004834.pub2 19370612

[41] Alireza Firooz (2006) Imiquimod in combination with meglumine antimoniate for cutaneous leishmaniasis: a randomized assessor-blind controlled trial. Arch Dermatol 142:1575–1579 17178983 10.1001/archderm.142.12.1575

[42] Sundar S, Murray HW (1995) Effect of Treatment with Interferon-γ Alone in Visceral Leishmaniasis. J Infect Dis 172:1627–1629 7594733 10.1093/infdis/172.6.1627

[43] Pin A (2019) An Easy and Reliable Strategy for Making Type I Interferon Signature Analysis Comparable among Research Centers. diagnostics (Basel) 9:113 31487897 10.3390/diagnostics9030113 PMC6787630

[44] Baechler EC (2003) Interferon-inducible gene expression signature in peripheral blood cells of patients with severe lupus. Proc Natl Acad Sci U S A 100:2610–2615 12604793 10.1073/pnas.0337679100 PMC151388

[45] Kim H (2018) Development of a Validated Interferon Score Using NanoString Technology. J Interf Cytokine Res 38:171–185 10.1089/jir.2017.0127 PMC5963606 29638206

[46] Alyass A, Turcotte M, Meyre D (2015) From big data analysis to personalized medicine for all: challenges and opportunities. BMC Med Genomics 8:33 26112054 10.1186/s12920-015-0108-y PMC4482045

[47] Giraldo-Parra L, Ramirez LG, Navas A, Gómez MA (2023) Quality parameters for RNA preparations from biopsies of ulcerated human skin. Wellcome Open Res 7:1–13 10.12688/wellcomeopenres.18052.1 PMC9984735 36879918

[48] Grimaldi G, McMahon-Pratt D (1996) Monoclonal antibodies for the identification of New World Leishmania species. Mem Inst Oswaldo Cruz 91:37–42 8734946 10.1590/s0074-02761996000100006

[49] Fernandez OL (2014) Miltefosine and antimonial drug susceptibility of Leishmania Viannia species and populations in regions of high transmission in Colombia. PLoS Negl Trop Dis 8:e2871 24853871 10.1371/journal.pntd.0002871 PMC4031164

[50] Fernández O (2012) Novel approach to in vitro drug susceptibility assessment of clinical strains of Leishmania spp. J Clin Microbiol 50:2207–2211 22518860 10.1128/JCM.00216-12 PMC3405580

[51] Bolger AM, Lohse M, Usadel B (2014) Trimmomatic: a flexible trimmer for Illumina sequence data. Bioinformatics 30:2114–2120 24695404 10.1093/bioinformatics/btu170 PMC4103590

[52] Kim D, Paggi JM, Park C, Bennett C, Salzberg SL (2019) Graph-based genome alignment and genotyping with HISAT2 and HISAT-genotype. Nat Biotechnol 37:907–915 31375807 10.1038/s41587-019-0201-4 PMC7605509

[53] Li H, Handsaker B, Wysoker A, Fennell T (2009) The Sequence Alignment/Map format and SAMtools. Bioinformatics 25:2078–2079 19505943 10.1093/bioinformatics/btp352 PMC2723002

[54] Anders S, Theodor Pyl P, Huber W (2015) HTSeq–a Python framework to work with high-throughput sequencing data. Bioinformatics 31:166–169 25260700 10.1093/bioinformatics/btu638 PMC4287950

[55] Leek JT, Johnson WE, Parker HS, Jaffe AE, Storey J (2012) D. The SVA package for removing batch effects and other unwanted variation in high-throughput experiments. Bioinformatics 28:882–883 22257669 10.1093/bioinformatics/bts034 PMC3307112

[56] Ritchie ME (2015) Limma powers differential expression analyses for RNA-sequencing and microarray studies. Nucleic Acids Res 43:1–13 25605792 10.1093/nar/gkv007 PMC4402510

[57] Robinson MD, McCarthy DJ, Smyth GK, edgeR (2010) A Bioconductor package for differential expression analysis of digital gene expression data. Bioinformatics 26:139–140 19910308 10.1093/bioinformatics/btp616 PMC2796818

[58] Love MI, Huber W, Anders S (2014) Moderated estimation of fold change and dispersion for RNA-seq data with DESeq2. Genome Biol 15:1–21 10.1186/s13059-014-0550-8 PMC4302049 25516281

[59] Leng N (2013) EBSeq: An empirical Bayes hierarchical model for inference in RNA-seq experiments. Bioinformatics 29:1035–1043 23428641 10.1093/bioinformatics/btt087 PMC3624807

[60] Kolberg L (2023) G:Profiler-interoperable web service for functional enrichment analysis and gene identifier mapping (2023 update). Nucleic Acids Res 51:W207–W212 37144459 10.1093/nar/gkad347 PMC10320099

[61] Hänzelmann S, Castelo R, Guinney J (2013) GSVA: gene set variation analysis for microarray and RNA-seq data. BMC Bioinformatics 14:7 23323831 10.1186/1471-2105-14-7 PMC3618321

[62] Szklarczyk D (2021) The STRING database in 2021: customizable protein-protein networks, and functional characterization of user-uploaded gene/measurement sets. Nucleic Acids Res 49:D605–D612 33237311 10.1093/nar/gkaa1074 PMC7779004

[63] Liberzon A (2011) Molecular signatures database (MSigDB) 3.0. Bioinformatics 27:1739–1740 21546393 10.1093/bioinformatics/btr260 PMC3106198

[64] Wood DE, Lu J, Langmead B (2019) Improved metagenomic analysis with Kraken 2. Genome Biol 20:257 31779668 10.1186/s13059-019-1891-0 PMC6883579

[65] Mehta CR, Patel NR (1983) A Network Algorithm for Performing Fisher’s Exact Test in r × c Contingency Tables. J Am Stat Assoc 78:427–434

